# Towards the Anaerobic Production of Surfactin Using *Bacillus subtilis*

**DOI:** 10.3389/fbioe.2020.554903

**Published:** 2020-11-26

**Authors:** Mareen Hoffmann, Diana Stephanie Fernandez Cano Luna, Shengbin Xiao, Lars Stegemüller, Katharina Rief, Kambiz Morabbi Heravi, Lars Lilge, Marius Henkel, Rudolf Hausmann

**Affiliations:** Department of Bioprocess Engineering (150k), Institute of Food Science and Biotechnology (150), University of Hohenheim, Stuttgart, Germany

**Keywords:** *Bacillus subtilis*, anaerobic cultivation, lipopeptide, surfactin, process control strategy, nitrate respiration, acetate, foam-free

## Abstract

The anaerobic growth of *B. subtilis* to synthesize surfactin poses an alternative strategy to conventional aerobic cultivations. In general, the strong foam formation observed during aerobic processes represents a major obstacle. Anaerobic processes have, amongst others, the distinct advantage that the total bioreactor volume can be exploited as foaming does not occur. Recent studies also reported on promising product per biomass yields. However, anaerobic growth in comparison to aerobic processes has several disadvantages. For example, the overall titers are comparably low and cultivations are time-consuming due to low growth rates. *B. subtilis* JABs24, a derivate of strain 168 with the ability to synthesize surfactin, was used as model strain in this study. Ammonium and nitrite were hypothesized to negatively influence anaerobic growth. Ammonium with initial concentrations up to 0.2 mol/L was shown to have no significant impact on growth, but increasing concentrations resulted in decreased surfactin titers and reduced nitrate reductase expression. Anaerobic cultivations spiked with increasing nitrite concentrations resulted in prolonged lag-phases. Indeed, growth rates were in a similar range after the lag-phase indicating that nitrite has a neglectable effect on the observed decreasing growth rates. In bioreactor cultivations, the specific growth rate decreased with increasing glucose concentrations during the time course of both batch and fed-batch processes to less than 0.05 1/h. In addition, surfactin titers, overall *Y*_P/X_ and *Y*_P/S_ were 53%, ∼42%, and ∼57% lower than in serum flask with 0.190 g/L, 0.344 g/g and 0.015 g/g. The *Y*_X/S_, on the contrary, was 30% lower in the serum flask with 0.044 g/g. The productivities *q* were similar with ∼0.005 g/(g⋅h). However, acetate strongly accumulated during cultivation and was posed as further metabolite that might negatively influence anaerobic growth. Acetate added to anaerobic cultivations in a range from 0 g/L up to 10 g/L resulted in a reduced maximum and overall growth rate μ by 44% and 30%, respectively. To conclude, acetate was identified as a promising target for future process enhancement and strain engineering. Though, the current study demonstrates that the anaerobic cultivation to synthesize surfactin represents a reasonable perspective and feasible alternative to conventional processes.

## Introduction

The cyclic lipopeptide surfactin synthesized by *Bacillus subtilis* is often described as a promising alternative to surfactants of petrochemical and oleochemical origin (Henkel et al., 2017) with additional antimicrobial properties (Ongena and Jacques, 2008; Li et al., 2019). However, conventional aerobic processes targeting at surfactin production share one major bottleneck, namely excessive foaming. The presence of foam in biotechnological processes often results, amongst others, in lower productivity (St-Pierre Lemieux et al., 2019). Excessive foaming may hinder probes to measure correctly, blocks exhaust air filters and hence pressure increases, and leads to heterogeneity in the cultivation broth (St-Pierre Lemieux et al., 2019). Different processes targeting surfactin production were reported that either integrate or avoid foaming. Integration of foaming is mostly performed as *in situ* product removal (Cooper et al., 1981; Davis et al., 2001; Chen et al., 2006; Willenbacher et al., 2014). Here, the ability of surfactin to accumulate at air-liquid interfaces is used as advantage and can be regarded as a first enrichment and purification step. Nevertheless, the uncontrolled foaming is reported to hinder process control and next to surfactin, also producer cells and the medium is lost for further cultivation (Willenbacher et al., 2014; Rangarajan and Clarke, 2015; Coutte et al., 2017). The membrane-bioreactor presented by Coutte et al. (2010) is an alternative foam-free cultivation strategy. This set-up yielded concentrations of 0.242 g/L surfactin. However, productivity was reduced during the time course of cultivation due to the adsorbance of surfactin onto the membranes which further reduced oxygen transfer. Chtioui et al. (2012) designed a rotating disk bioreactor where surfactin was produced by *B. subtilis* ATCC 21332 both in free cells and cells immobilized as a biofilm on the rotating disks. Aeration was performed above the liquid level and was reported to not be sufficient and surfactin concentrations did not surpass 0.212 g/L. Another strategy to synthesize surfactin was illustrated by Davis et al. (1999). Different batch cultivations with e.g., nitrate-limitation, carbon-limitation or oxygen-limitation demonstrated that highest specific product yield per biomass (*Y*_P/X_) was achieved in nitrate-limited oxygen-depleted cultures. The authors reported that anaerobic growth occurred in oxygen-depleted conditions. However, aeration was still maintained at 0.5 vvm indicating the presence of microaerophilic conditions. This strategy was further adapted by Willenbacher et al. (2015a) employing strain *B. subtilis* DSM 10^T^. Anaerobic conditions were obtained as aeration was completely avoided, which also resulted in a foam-free environment without the need of adding antifoam. This process reached high values for the product yield per biomass *Y*_P/X_ with 0.278 g/g employing 2.5 g/L glucose. The anaerobic cultivation takes advantage of the ability of *B. subtilis* to use nitrate as alternative electron acceptor in the absence of oxygen. During nitrate respiration, nitrate is reduced to ammonium via nitrite using the enzymes nitrate reductase NarGHI and nitrite reductase NasDE (Nakano et al., 1998a). A recent study demonstrated that anaerobic serum flask cultivations employing strain *B. subtilis* JABs24, which is the well-established laboratory strain 168 with the ability to synthesize surfactin due to integration of a functional *sfp* gene, reached excellent values for *Y*_P/X_ with 1.541 g/g and these values surpassed aerobic results (Geissler et al., 2019b). Next to the foam-free environment that can be achieved employing anaerobic cultivations, another advantage is the more than hundred thousand times higher solubility of nitrate compared to oxygen in the medium. This allows for more flexibility in the design of batch and fed-batch processes. Furthermore, the development of nitrate respiration processes might generally be beneficial for products sensitive against oxidation. However, recent studies of both Willenbacher et al. (2015a) and Geissler et al. (2019b) have reported a much lower cell dry weight accompanied by overall inferior surfactin titers compared to aerobic counterparts. As a consequence, this study aimed at further evaluating the relevance of nitrite and ammonium as well as the impact of glucose concentrations for an envisioned foam-free anaerobic surfactin bioproduction process.

We hypothesized that the presence of both nitrite and ammonium has a negative impact on anaerobic nitrate respiration, while different initial glucose concentrations play a minor role. To further substantiate this hypothesis, reporter strains carrying P*_*narG*_-lacZ* and P*_*nasD*_-lacZ* fusion were included to evaluate effects on the most important enzymes during anaerobic nitrate respiration.

## Materials and Methods

### Chemicals and Materials

All chemicals used were of analytical grade and were purchased from Carl Roth GmbH & Co., KG (Karlsruhe, Germany). The surfactin reference standard (≥98% purity) and glucose standard were obtained from Sigma-Aldrich Laborchemikalien GmbH (Seelze, Germany).

### Microorganisms, Genetic Engineering and Strain Maintenance

All strains used within this study are listed in Table 1. Strain *B. subtilis* JABs24, constructed as described in Geissler et al. (2019b), is derived from the laboratory strain 168 with functional *sfp* and was used as initial strain for the construction of the reporter strains MG1 and MG5. The oligonucleotides (Eurofins Genomics Germany GmbH, Ebersberg, Germany) and plasmids used are listed in Supplementary Table S1 and Table 2, respectively. The promoter regions of *narG* and *nasD* were amplified through PCR (peqSTAR 96X, VWR GmbH, Darmstadt, Germany) using primers s1001/s1002, and s1011/s1012, respectively. The PCR products were purified (GeneMATRIX basic DNA purification Kit, EURx Sp. Z o.o, Gdańsk, Poland) and ligated into plasmid pJOE4786.1 (Altenbuchner et al., 1992) with T4 DNA ligase (New England BioLabs GmbH, Frankfurt am Main, Germany) after digestion with *Sma* I (New England BioLabs GmbH, Frankfurt am Main, Germany). The obtained plasmids, pKAM0182 for P*_*narG*_* and pSHX1 for P*_*nasD*_*, were transformed into chemical competent *E. coli* JM109. Strains carrying the plasmid were selected on LB plates supplemented with ampicillin (100 μg/mL). Isolated plasmids pKAM0182 and pSHX1 (QIAamp DNA Mini Kit (50), QIAGEN GmbH, Hilden, Germany) were digested with *Nde* I and *Age* I (New England BioLabs GmbH, Frankfurt am Main, Germany) and fragments of interest were isolated (MinElute Gel Extraction Kit (50), QIAGEN GmbH, Hilden, Germany). Afterwards, digestion products were ligated into pKAM312 (Morabbi Heravi and Altenbuchner, 2018) resulting in pKAM452 and pSHX2 and transformed in competent *E. coli* JM109. Plasmids pKAM452 (P*_*narG*_-lacZ*) and pSHX2 (P*_*nasD*_-lacZ*) were isolated and were transformed into natural competent *B. subtilis* JABs24. By double cross-over into the *amyE* gene, reconstructed strains were selected by agar plates supplemented with either spectinomycin (100 μg/mL) or erythromycin (10 μg/mL), and by starch plates dyed with Lugol’s iodine solution. Positive colonies were further checked by PCR using primers s7406 and s7409 to confirm insertion of P*_*narG*_-lacZ* and P*_*nasD*_-lacZ* into *amyE* gene.

**TABLE 1:** Overview of strains used in the current study.

Strain	Genotype or description	References
***B. subtilis***		
JABs24	*trp*^+^ *sfp*^+^ Δ*manPA*	Geissler et al. (2019b)
MG1	*trp*^+^ *sfp*^+^ Δ*manPA amyE::[*P*_*narG*_-lacZ, spcR]*	This study
MG5	*trp*^+^ *sfp*^+^ Δ*manPA amyE*::[P*_*nasD*_-lacZ, spcR*]	This study
***E. coli***		
JM109	*mcrA recA1 supE44 endA1 hsdR17 (r_*K*_^–^m_*K*_^+^) gyrA96 relA1 thi Δ(lac-proAB) F′* [*traD36 proAB^+^ lacI^*q*^ lacZ ΔM15*]	Yanisch-Perron et al. (1985)

**TABLE 2:** Plasmids used in this study.

Plasmid	Properties or insert	References
pJOE4786.1	*ori*_*pUC18*_, *bla*, *ter*-′*lacI-lacZα-ter*	Altenbuchner et al. (1992)
pKAM0182	pJOE4786.1 + PCR s1001 – s1002 (*Sma* I)	This study
pSHX1	pJOE4786.1 + PCR s1011 – s1012 (*Sma* I)	This study
pKAM312	*ori*_*pBR322*_, *rop*, *ermC*, *bla*, *amyE*′-[*ter*-P*_*glcR*_-lacZ-spcR*]-′*amyE*	Morabbi Heravi and Altenbuchner (2018)
pKAM452	pKAM312 containing promoter	This study
	region of *narG* (pKAM0182), integrated by *Age* I and *Nde* I	
pSHX2	pKAM312 containing promoter	This study
	region of *nasD* (pSHX1), integrated by *Age* I and *Nde* I	

### Media Composition

The LB medium used for the first pre-culture composed of 5 g/L tryptone, 10 g/L NaCl and 10 g/L yeast extract. An adapted medium based on the medium described by Willenbacher et al. (2015b) was used for all further pre-cultures and main cultures. The glucose concentration was 20 g/L for the second mineral salt pre-culture, 10 g/L and 2.5 g/L glucose for the batch and fed-batch bioreactor process, as well as either 2.5, 5, 7.5, or 10 g/L glucose for the serum flask cultivations as indicated in the respective results. The buffer composed of 0.03 mol/L KH_2_PO_4_ and 0.04 mol/L Na_2_HPO_4_ ⋅ 2 H_2_O in the mineral salt pre-culture and serum flask cultivations, and 5.71 ⋅ 10^–3^ mol/L KH_2_PO_4_ and 4.29 ⋅ 10^–3^ mol/L Na_2_HPO_4_ ⋅ 2 H_2_O in the bioreactor cultivations. The nitrogen source used for the mineral salt pre-culture was 0.1 mol/L NaNO_3_ and for the bioreactor and serum flask cultivation 0.1 mol/L NaNO_3_ and 5.0 ⋅ 10^–4^ mol/L (NH_4_)_2_SO_4_. In case of MgSO_4_ and trace element solution, which were prepared separately as autoclaved, respectively, filter sterilized stock solutions, all cultivations had the same final concentrations with 8.0 ⋅ 10^–6^ mol/L Na_3_-citrate, 7.0 ⋅ 10^–6^ mol/L CaCl_2_, 4.0 ⋅ 10^–6^ mol/L FeSO_4_ ⋅ 7 H_2_O, 1.0 ⋅ 10^–6^ mol/L MnSO_4_ ⋅ H_2_O, 4.0 ⋅ 10^–6^ mol/L Na_2_MoO_4_ ⋅ 2 H_2_O and 8.0 ⋅ 10^–4^ mol/L MgSO_4_ ⋅ 7 H_2_O. A 25% (*w*/*w*) autoclaved (121°C, 20 min) glucose solution was used for the bioreactor feed. For the cultivations investigating the effect of ammonium, the concentration of (NH_4_)_2_SO_4_ was adjusted. In case of nitrite and acetate experiments, the targeted concentrations were added from an autoclaved 0.1 mol/L NaNO_2_ or 277.88 g/L Na-acetate stock solution, respectively.

### Cultivation Conditions

Pre-cultures were run at 120 rpm and 37°C in an incubator shaker (NewbrunswickTM/Innova 44, Eppendorf AG, Hamburg, Germany). A first overnight pre-culture was performed in a 100 mL baffled shake flask by inoculating 20 mL LB medium with 100 μL of the respective glycerol stock. This pre-culture was diluted 1:100 in mineral salt medium for a second pre-culture and incubated for 36 h. The shake flask size was adjusted to the amount of inoculation material needed and flasks were filled with 10–13% of the mineral salt medium.

### Anaerobic Serum Flask Cultivation

Anaerobic serum flasks were prepared as described in Geissler et al. (2019b). Briefly, the buffer and nitrogen sources were autoclaved inside the flasks and all other solutions were added afterwards through a disinfected septum using a syringe. Anaerobic conditions were set by flushing the flasks with nitrogen and degassing through a filter element.

### Anaerobic Bioreactor Cultivation

Bioreactor cultivations were performed in 42 L custom-built bioreactors (ZETA GmbH, Graz/Lieboch, Switzerland). The bioreactors are mounted on a scale and are equipped with pH (EasyFerm Bio HB Arc 120, Hamilton Bonaduz AG, Bonaduz, Switzerland) and pO_2_ (VisiFerm DO ARC 120 H0, Hamilton Bonaduz AG, Bonaduz, Switzerland) probes. Acid, base and feed solutions were on individual scales and added via peristaltic pumps. Bioreactors were equipped with three Rushton turbines and four baffle plates. The buffer and nitrogen source were autoclaved inside the bioreactor and the other components were added sterile through a septum. Prior to inoculation, the medium was flushed with N_2_ to ensure anaerobic conditions and pO_2_ measurement throughout cultivation confirmed absence of oxygen. Stirrer speed was kept at 120 rpm and pH 7 was maintained by adding either 1 mol/L NaOH or 1 mol/L H_3_PO_4_. Temperature was set to 37°C.

### Sampling and Sample Analysis

For all cultivations performed, samples were taken in regular intervals. The OD_600_ was measured with a spectrophotometer (Biochrom WPA CO8000, Biochrom Ltd., Cambridge, United Kingdom). The cell dry weight was calculated by dividing the OD_600_ by the factor 3.762 which was determined previously (Geissler et al., 2019b). Prior to further analyses, samples were centrifuged for 10 min at 4°C and 4816 *g* (Heraeus X3R, Thermo Fisher Scientific GmbH, Braunschweig, Germany) and stored at −20°C until further processing.

Glucose was measured with a HPTLC system (CAMAG AG, Muttenz, Switzerland) as described in Geissler et al. (2019b). Briefly, the mobile phase used was acetonitrile/H_2_O (85:15, *v*/*v*) and plates were developed over a migration distance of 70 mm. After development, plates were derivatized with diphenylamine (DPA) reagent. DPA was prepared by diluting 2.4 g diphenylamine and 2.4 g aniline in 200 mL methanol and then adding 20 mL 85% phosphoric acid. After derivatization, plates were scanned at 620 nm and the glucose concentration was calculated in dependence of the standard curve.

Surfactin analysis was performed as described in Geissler et al. (2017) using a HPTLC method. Briefly, samples were extracted three times with an equal volume of chloroform:methanol 2:1 (*v*/*v*). The pooled solvent layers were evaporated and the crude surfactin was resuspended in methanol to match the initial sample volume. Plates were developed using the mobile phase chloroform:methanol:water 65:25:4 (*v*/*v*/*v*) over a migration distance of 60 mm. After development, the plates were scanned at 195 nm and evaluation was performed by peak area in correspondence to a standard curve.

Spectrophotometric assays (Merck KGaA, Darmstadt, Germany) were used to measure nitrate (Cat. No. 1.09713.0001), nitrite (Cat. No. 1.14776.0001) and ammonium (Cat. No. 1.14752.0001) concentrations.

Acetate concentration was determined with an enzymatic kit (Cat. No. 10148261035, r-biopharm AG, Pfungstadt, Germany).

For ß-galactosidase assay, a volume of 100 μL of cell suspension from strain MG1 or MG5 was mixed with 900 μL Z-Buffer (0.06 mol/L Na_2_HPO_4_, 0.04 mol/L NaH_2_PO_4_, 0.01 mol/L KCl, 1 mmol/L MgSO_4_ ⋅ 7 H_2_O, 0.04 mol/L mercaptoethanol). After addition of 10 μL toluol, the mixture was incubated for 30 min at 37°C and 750 rpm. 200 μL of 20 mmol/L *ortho*-nitrophenylgalactopyranoside was added and the reaction was stopped when the solution turned yellow by adding 500 μL of 1 mol/L Na_2_CO_3_. Samples were centrifuged for 2 min at 19283 *g* and 250 μL were transferred to a microtiter plate. Absorbance was measured at both 420 nm and 550 nm and the Miller Units (MU) were calculated according to the following equation:

$$\text{MU}=1000\cdot \frac{\left({\text{OD}}_{420\,\mathrm{nm}}-\left(1.75\cdot {\text{OD}}_{550\,\mathrm{nm}}\right)\right)}{t\cdot \upsilon \cdot {\text{OD}}_{600\,\mathrm{nm}}}$$

### Data Analysis

For the bioreactor cultivations performed, as well as for serum flask cultivations if required, the biomass yield per substrate *Y*_X/S_ [g/g], product yield per substrate *Y*_P/S_ [g/g], the product yield per biomass *Y*_P/X_ [g/g], the specific surfactin productivity *q* [g/(g⋅h)] and the specific growth rate μ [1/h] were determined. The respective equations are listed in the Supplementary Material (S2). Depending on the evaluation, either surfactin or acetate was considered as product P. These parameters were calculated in two distinct approaches. Maximum yields were determined by calculating the respective parameter in between sampling points and overall yields were calculated based on the data of inoculation and at CDW_max_ of the process. For the bioreactor cultivation employing a feed, the glucose fed at sampling was added to the respective time point.

## Results and Discussion

### Influence of Different Ammonium Concentrations on Anaerobic Growth and Effect on Promoter Activity of P_*narG*_ and P_*nasD*_

Under aerobic conditions, ammonium is the preferred nitrogen source and in the presence of both ammonium and nitrate, nitrate consumption is induced after depletion of ammonium (Davis et al., 1999). During anaerobic nitrate respiration, however, nitrate is used as alternative electron acceptor and is thereby reduced to nitrite which is further reduced to ammonium by the enzymes nitrate reductase NarGHI and nitrite reductase NasDE, respectively (Hoffmann et al., 1998; Nakano et al., 1998a; Härtig and Jahn, 2012). As these enzymes are crucial for anaerobic nitrate respiration, the corresponding gene expressions were monitored by the respective promoters P*_*narG*_* and P*_*nasD*_*. As the concentration of ammonium is expected to increase during cultivation due to nitrate reduction, it was hypothesized that the increase in ammonium might have a negative impact on both enzyme activity as well as gene expression, the latter one being studied using the reporter strains. In addition, a low initial ammonium concentration was hypothesized to be sufficient as ample pool for the incorporation of ammonium into biomass until ammonium is provided by nitrate respiration. In this sense, the influence of different ammonium concentrations on the growth of *B. subtilis* under anaerobic conditions was examined. In a first screening, *B. subtilis* JABs24 was cultivated in duplicate employing 2.5 g/L glucose with five various ammonium concentrations ranging from 0.001 mol/L NH_4_^+^ up to 0.2 mol/L NH_4_^+^. The overall growth rates μ were in the range of 0.068 ± 0.006 1/h. Also with respect to the final CDW, which was in the range of 0.194 ± 0.015 g/L, and the time to reach CDW_max_, an influence of the ammonium concentration was not observed indicating that a high initial ammonium concentration as well as the increase in ammonium due to nitrate respiration did not negatively influence anaerobic growth when employing 2.5 g/L glucose. However, as reported by Willenbacher et al. (2015a), an increase in the initial glucose concentration from 7.5 g/L to 10 g/L resulted in a lower CDW and *Y*_X/S_ employing strain *B. subtilis* DSM 10^T^. The initial NH_4_^+^ concentration in this study was 0.1 mol/L. Assuming a complete conversion from nitrate to ammonium, final concentrations were about 0.16 and 0.14 mol/L NH_4_^+^ for 7.5 and 10 g/L glucose, respectively. In this sense, further cultivations employing the reporter strains *B. subtilis* MG1 (P*_*narG*_-lacZ*) and MG5 (P*_*nasD*_-lacZ*) with both 7.5 and 10 g/L glucose, as well as 0.001 mol/L, 0.1 mol/L, and 0.2 mol/L NH_4_^+^ were further used to examine the combinative effect of different ammonium and glucose concentrations on anaerobic growth by nitrate respiration.

**FIGURE 1:**
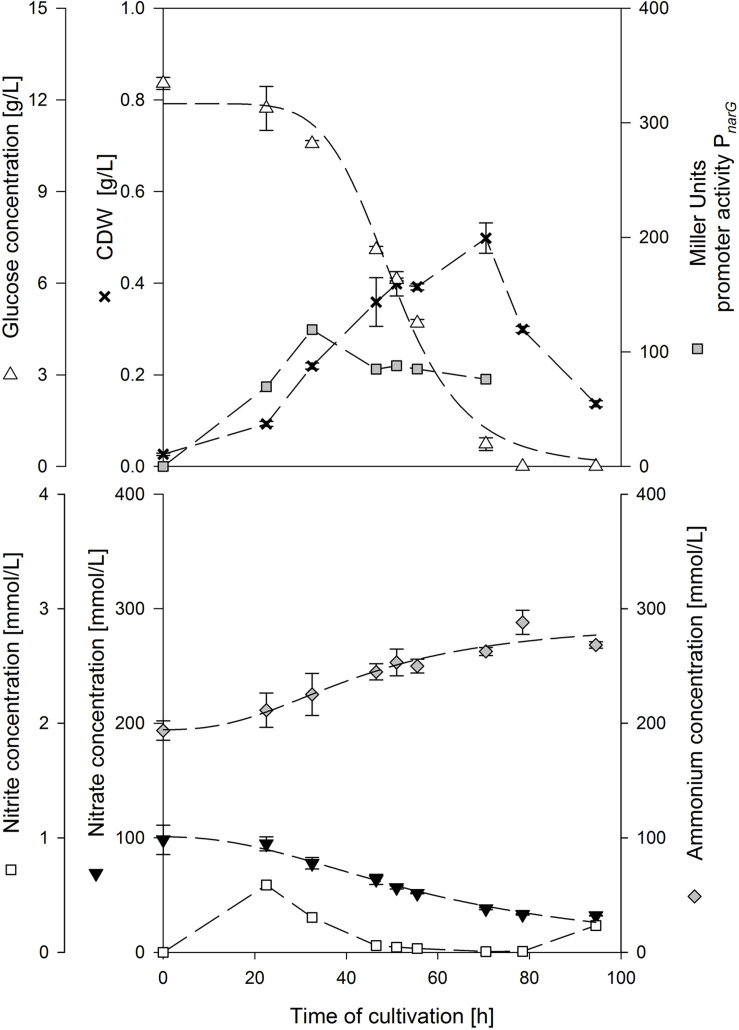
Exemplary anaerobic serum flask cultivation employing strain *B. subtilis* MG1 in a mineral salt medium containing 10 g/L glucose, 0.1 mol/L NO_3_^–^ and 0.2 mol/L NH_4_^+^.

Figure 1 displays an exemplary cultivation plot of strain MG1 employing 10 g/L glucose and 0.2 mol/L NH_4_^+^. The biomass increased up to 0.5 g/L after 70.5 h of cultivation. Glucose was almost depleted when CDW_max_ was reached and hence the drop in CDW_max_ was caused by glucose depletion. About 70 mmol/L NO_3_^–^ was reduced and ammonium increased by ∼60 mmol/L. Nitrite peaked at the beginning of cultivation to 0.558 mmol/L and was further reduced to 0.007 mmol/L at CDW_max_. Another increase was measured when CDW decreased. This nitrite pattern was observed in almost all cultivations tested. The activity of P*_*narG*_* increased up to 119 MU after 32.5 h of cultivation and slightly decreased to 76 MU until CDW_max_. For a better evaluation, the results of the different ammonium and glucose concentrations tested are summarized in Table 3.

**TABLE 3:** Summary of the results and calculated yields for the serum flask cultivations of strains *B. subtilis* MG1 (P*_*narG*_-lacZ*) and MG5 (P*_*nasD*_-lacZ*) employing 7.5 g/L and 10 g/L glucose and different ammonium concentrations.

	Strain	7.5 g/L glucose			10 g/L glucose		
		0.001 mol/L NH_4_^+^	0.1 mol/L NH_4_^+^	0.2 mol/L NH_4_^+^	0.001 mol/L NH_4_^+^	0.1 mol/L NH_4_^+^	0.2 mol/L NH_4_^+^
CDW_max_ [g/L]	MG1	0.2990.033	0.3190.013	0.4850.033	0.4590.007	0.3790.007	0.4980.033
	MG5	0.3990.040	0.3920.007	0.4650.027	0.3320.093	0.4050.020	0.4250.040
Overall μ [1/h]	MG1	0.0500.004	0.0610.001	0.0760.004	0.0440.008	0.0340.002	0.0420.002
	MG5	0.0590.002	0.0670.002	0.0620.003	0.0550.002	0.0450.001	0.0510.009
*Y*_X/S_ [g/g]	MG1	0.0400.001	0.0440.004	0.0780.012	0.0370.003	0.0290.001	0.0400.003
	MG5	0.0560.004	0.0810.001	0.0580.003	0.0480.004	0.0360.002	0.0520.008
Surfactin [mg/L]	MG1	109.973.23	94.552.00	83.197.37	156.203.46	136.404.40	87.353.99
	MG5	85.5313.41	85.613.97	48.492.02	74.9017.95	66.4215.35	32.637.82
maximum Miller Units	MG1	238.373.08	156.518.88	115.902.90	231.709.16	152.0725.33	119.458.24
	MG5	669.2935.62	692.4844.51	666.4616.74	779.2420.70	982.14196.30	708.5678.74
Miller Units at CDW_max_	MG1	176.5730.27	145.768.51	77.120.45	127.070.95	116.231.85	76.214.02
	MG5	605.2813.14	543.5238.63	569.5847.52	740.50173.45	746.76136.21	559.6370.19

A higher glucose concentration and especially the presence of 0.2 mol/L NH_4_^+^ within the same glucose level resulted in a higher CDW_max_. Discrepancies, however, must be considered as the determined CDW_max_ in several cultivations was prior to glucose depletion or even slightly after glucose depletion due to the time intervals of sampling. A CDW_max_ of 0.388 g/L was reported by Geissler et al. (2019b) employing strain *B. subtilis* JABs24 using 10 g/L glucose, 0.1 mol/L NO_3_^–^ and 0.025 mol/L NH_4_^+^. This value is in a similar range in comparison to this study. For strain MG1 with 7.5 g/L, both the *Y*_X/S_ and the overall growth rate μ increased when more ammonium was added to the medium. For all other cultivations, this trend for both the overall growth rate μ and the *Y*_X/S_ was not observed. Hence, no generally admitted influence of ammonium could be observed at these experimental conditions, which is also in agreement to the results employing 2.5 g/L glucose. Under aerobic conditions, for example, Leejeerajumnean et al. (2000) reported that 26 tested *Bacillus* strains, among them strain *B. subtilis* NCIMB 3610, grew in the presence of 931 mmol/L NH_4_^+^ at pH 7. Müller et al. (2006) described that 500 mmol/L did not cause growth inhibition using strain *B. subtilis* 168. A defect was observed with more than 750 mmol/L NH_4_^+^, but the authors stated that this is due to osmotic or ionic stress and not due to the presence of ammonium itself. However, no reports on the effect of ammonium under anaerobic conditions were found.

Interestingly, the surfactin concentration was lower the more ammonium was present at the beginning of cultivation, with a more drastic change in between 0.1 mol/L and 0.2 mol/L NH_4_^+^. Exemplary, employing 10 g/L glucose and 0.001 mol/L NH_4_^+^ resulted in a surfactin concentration of 156.20 mg/L, while the titer obtained with 0.2 mol/L NH_4_^+^ was only 87.35 mg/L for strain MG1. On the contrary, when the ratio of NO_3_^–^:NH_4_^+^ was shifted towards higher nitrate concentrations in studies on the surfactin synthesis under aerobic conditions, a decrease in biomass and surfactin concentration, but an increase in the *Y*_P/X_ was observed. For example, media containing only NH_4_^+^ or NO_3_^–^ resulted in a surfactin titer of ∼1 g/L and ∼0.35 g/L at an CDW_max_ of 2 g/L after 21 h and of 0.5 g/L after 36 h of cultivation with strain *B. subtilis* JABs24, respectively. This results in *Y*_P/X_ values of 0.466 g/g and 0.668 g/g (data not shown). On the contrary, Davis et al. (1999) reported on an improvement in the *Y*_P/X_ when cultivating *B. subtilis* ATCC 21332 in a nitrate-limited oxygen-depleted process. Consequently, further medium optimization studies on the impact of different nitrate concentrations at constant ammonium levels on surfactin synthesis are a crucial approach also to investigate the impact on both the dissimilatory and assimilatory nitrogen metabolism.

With respect to the promoter activities, averaged maximum Miller Units for P*_*narG*_* were overall lower with ∼170 MU than for P*_*nasD*_* with ∼750 MU. During the time course of cultivation, as illustrated in Supplementary Figure S3 and summarized in Table 3, the promoter activity decreased after reaching the maximum until CDW_max_. For P*_*narG*_*, both the maximum promoter activity as well as the activity at CDW_max_ was lower the more ammonium was present. Differences amongst the glucose concentrations tested were less distinct. Hence, the impact of glucose was less significant than the ammonium concentration and lower ammonium concentrations yielded a higher P*_*narG*_* activity. The activity of P*_*nasD*_* was overall much higher with Miller Units up to 1000, but the pattern was similar to P*_*narG*_* and activity reached a maximum and further declined until CDW_max_. For the P*_*nasD*_* activity, values at 10 g/L glucose were overall higher in comparison to the respective data at 7.5 g/L glucose. However, highest values were obtained for 0.1 mol/L NH_4_^+^. Hence, a trend was not observed regarding the influence of increasing ammonium concentrations. This is in agreement to the results of Nakano et al. (1998a) reporting that the presence of ammonium did not alter the anaerobic expression levels of a transcriptional fusion *nasD-lacZ* strain.

Generally, in the current experimental set-up, the increase of ammonium showed a tendency to an overall improvement with respect to CDW_max_ while a distinct trend for growth rate μ, *Y*_X/S_ and promoter activity P*_*nasD*_* was not observed. However, a decrease in both P*_*narG*_* activity and surfactin synthesis was noticed. Due to this ambiguous effect of ammonium on growth but the negative effect on surfactin synthesis and P*_*narG*_* activity, an initial ammonium concentration of 0.001 mol/L was used for all further experiments, as surfactin is the product of interest. In addition, as a further increase in CDW during anaerobic growth beyond 10 g/L glucose will result in a further accumulation of ammonium, the reduction to a minimum from the beginning is expected to be more profitable at long-term view.

### Influence of Different Nitrite Concentrations on Anaerobic Growth

As previously described and illustrated in Figure 1, nitrite concentrations peaked shortly after inoculation and decreased during the time course of further cultivation. On the one hand, nitrite is often stated to be toxic and hence cells need to detoxify accumulated nitrite, on the other hand, the reduction to ammonium by nitrite reductase is an electron sink which allows the reoxidation of NADH to NAD^+^ (Cruz Ramos et al., 1995; Nakano et al., 1998a; Reents et al., 2006). NAD^+^ itself is needed for glycolysis, oxidative decarboxylation and the citric acid cycle. Inversely, in the absence of nitrate or nitrite as electron acceptor, *B. subtilis* growth by fermentation and NAD^+^ would be regenerated through end product phosphorylation, with the main fermentative metabolites produced being lactate, acetate and 2,3-butandiol (Cruz Ramos et al., 2000).

**FIGURE 2:**
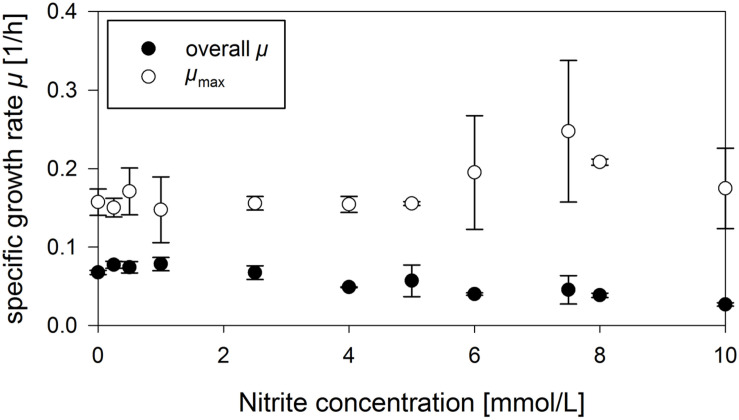
Influence of different nitrite concentrations on the overall and maximum specific growth rate μ in anaerobic serum flask cultivations employing strain *B. subtilis* JABs24.

To further elucidate the impact of nitrite on anaerobic growth, the influence of various concentrations in the range from 0 mmol/L up to 20 mmol/L on the growth behavior of strain JABs24 employing 2.5 g/L glucose was investigated. In the time frame cultivated, the overall growth rate was reduced which was basically due to an increase in lag-phase. For 8 mmol/L and 6 mmol/L NO_2_^–^, strains restored growth after a lag-phase of around 30 h. With lower nitrite concentrations of 4, 2.5, and 1 mmol/L, growth was detected after a lag-phase of 26, 24, and 12 h of cultivation, respectively. These results are further affirmed by the overall growth rate and maximum growth rate, illustrated in Figure 2. A decrease in the overall growth rate μ from 0.068 1/h to 0.027 1/h was observed for 0 mmol/L and 10 mmol/L NO_2_^–^ added in the time window tested. For the maximum growth rates, mean values varied in between 0.141 1/h and 0.208 1/h. However, higher nitrite concentrations did not result in lower growth rates. This is also in agreement to literature, where anaerobic growth of different *B. subtilis* strains employing either 10 mmol/L nitrate or nitrite resulted in the same OD-values indicating the suitability of nitrite as alternative electron acceptor (Hoffmann et al., 1998; Cruz Ramos et al., 2000; Marino et al., 2001). Interestingly, authors did not report on an increased lag-phase as observed in this study. For 20 mmol/L NO_2_^–^ and 2.5 g/L glucose, growth was not detected during the current experiment. To further investigate the effect of high nitrite concentrations and a longer time frame, a further cultivation employing 10 g/L glucose with both 10 and 20 mmol/L NO_2_^–^ was performed. For 20 mmol/L, growth was not detected even after 90 h of cultivation. For 10 mmol/L NO_2_^–^, CDW values up to 0.5 g/L were measured after ∼115 h. Compared to the cultivations employing different ammonium concentrations, the time to reach CDW_max_ was hence almost twice as long. However, the final CDW was comparably high and the concentration of nitrite was also drastically reduced until CDW_max_ was reached (data not shown).

To sum up the effect of nitrite, only at very high levels, nitrite is indeed growth limiting or even inhibiting. Concentrations up to 10 mmol/L did significantly increase the time of cultivation employing strain *B. subtilis* JABs24, but the maximum growth rates μ as well as CDW_max_ prior to glucose depletion were similar to the references without further additives and nitrite was also reduced to ammonium.

### Impact of Various Glucose Concentrations on Anaerobic Growth and Effect on Promoter Activity of P_*narG*_ and P_*nasD*_

During the study of Willenbacher et al. (2015a) using strain *B. subtilis* DSM 10^T^, a benefit of employing 10 g/L glucose with respect to higher CDW and surfactin concentrations was not given. Willenbacher et al. (2015a) stated that a concentration of 10 g/L glucose leads to overflow metabolism in *B. subtilis*. However, no data regarding this hypothesis, such as acetate concentrations, were shown. The previous results of cultivations with strain *B. subtilis* JABs24 and various ammonium concentrations indicated that the effect of 7.5 g/L and 10 g/L is less severe than reported by Willenbacher et al. (2015a) considering the CDW_max_, but an increase in glucose indeed decreased overall growth rates μ and resulted in lower *Y*_X/S_ values (Table 3). As the glucose concentration plays a major role in the design of batch and fed-batch processes, cultivations with 2.5, 5, 7.5, and 10 g/L glucose were performed in serum flasks employing the strains *B. subtilis* JABs24, MG1 and MG5 as one triplicate set-up.

**FIGURE 3:**
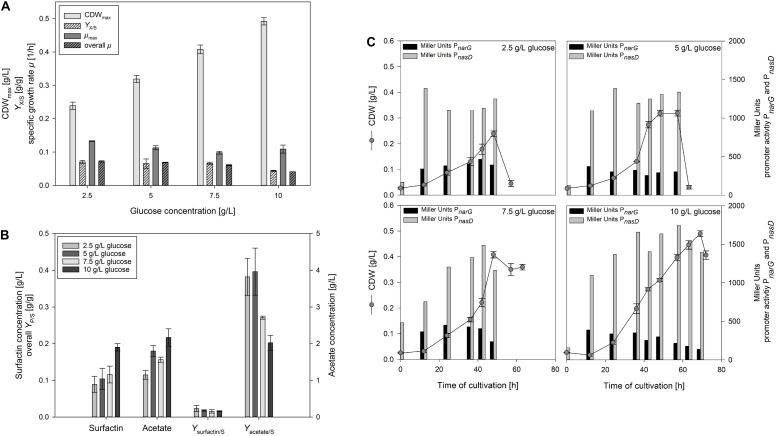
Influence of different glucose concentrations in anaerobic *B. subtilis* JABs24, MG1 and MG5 serum flask cultivations on **(A)** CDW_max_, biomass per substrate yield *Y*_X/S_, specific maximum and overall growth rate μ and **(B)** surfactin and acetate concentrations at CDW_max_, surfactin per substrate yield *Y*_surfactin/S_ and acetate per substrate yield *Y*_acetate/S_. In addition, **(C)** CDW and corresponding Miller Units measured for expression of promoters P*_*narG*_* and P*_*nasD*_* during the time course of cultivation.

Figure 3A illustrates the change in CDW_max_, *Y*_X/S_ as well as μ_max_ and overall μ of the serum flask cultivations employing different glucose concentrations. CDW_max_ increased by employing higher glucose concentrations from 0.239 ± 0.011 g/L up to 0.492 ± 0.011 g/L. In addition, the time to reach CDW_max_ increased from 30 h to 69 h the higher the glucose concentration was. The observation from Willenbacher et al. (2015a) could consequently not be confirmed and higher cell densities were actually reached the more glucose was added. However, it must be emphasized that different strains were used and in addition, even a frequent sampling does not assure to measure the CDW when glucose is about to be depleted, which was also reported by Geissler et al. (2019b). For the overall growth rate μ, a decrease from 0.072 ± 0.002 1/h to 0.041 ± 0.000 1/h was observed with increasing glucose concentration. In contrast, the differences in μ_max_ and the *Y*_X/S_ were less distinct. For μ_max_, values ranged from 0.098 ± 0.004 1/h to 0.133 ± 0.001 1/h, and for the overall *Y*_X/S_ between 0.044 ± 0.002 g/g and 0.070 ± 0.005 g/g.

Figure 3B displays the surfactin and acetate concentrations at CDW_max_ as well as the corresponding overall *Y*_P/S_. The surfactin concentration employing 10 g/L glucose was ∼2-fold higher with 189.72 ± 10.50 mg/L than at the other concentrations tested. On the contrary, maximum surfactin titers at the other glucose levels tested were in a similar range and only a slight trend towards an increase in surfactin at higher glucose levels was monitored. The *Y*_surfactin/S_ showed a similar trend than the *Y*_X/S_ and decreased with increasing glucose concentrations from 0.023 ± 0.008 g/g to 0.016 ± 0.001 g/g. As reported by Willenbacher et al. (2015a), authors assumed that overflow metabolism led to their results employing 10 g/L glucose. In the current study, acetate concentrations were measured for the samples at CDW_max_. Acetate is reported to be the most abundant end product during anaerobic growth of *B. subtilis* (Cruz Ramos et al., 2000). The acetate concentrations produced during anaerobic growth increased from 1.15 ± 0.12 g/L to 2.17 ± 0.24 g/L with increasing glucose concentrations. The *Y*_acetate/S_ was more than 10-fold higher than the *Y*_surfactin/S_ and decreased as well with increasing glucose concentration from 0.382 ± 0.051 g/g to 0.202 ± 0.020 g/g. This result is, however, rather counter intuitive, as higher glucose concentrations actually do not result in more acetate per glucose. Although further fermentative end products were not expected as nitrate respiration is reported to suppress fermentative growth (Cruz Ramos et al., 2000), lactate was measured as well. As assumed, at CDW_max_ employing 10 g/L glucose the lactate concentration reached 0.24 ± 0.01 g/L, and 0.16 ± 0.01 g/L employing 7.5 g/L glucose. This indicates that the glucose flux, in contrast to acetate, into lactate is comparably low, but that a high amount of glucose converts into acetate and not to the target product. Nakano et al. (1998b) reported that several enzymes of the citric acid cycle show a reduced activity anaerobically and authors assumed that citrate deficiency might cause *citZ* repression. In this sense, carbon flux studies to investigate glucose degradation are another interesting option for future studies.

Similarly to the previous cultivations, the effect of glucose on anaerobic nitrate respiration was also investigated by the inclusion of the reporter strains MG1 and MG5. Figure 3C illustrates the growth curves as well as the corresponding measured promoter activities for both P*_*narG*_* and P*_*nasD*_*. For P*_*narG*_*, the activity at CDW_max_ was lower with increasing glucose concentrations and decreased from 392 MU to 137 MU. However, MU values in the early stages of cultivation also reached highest MU values in between 370 – 440 MU. Hence, the promoter activity of the nitrate reductase was lower the longer the cultivation lasted. In combination with the previous results (Table 3), both high glucose as well as high ammonium concentrations decreased the P*_*narG*_* activity, but further studies have to address the question if actually the decreasing concentration of nitrate has an impact on P*_*narG*_* activities. In accordance to the previous results, the activity of P*_*nasD*_* was much higher with MU values up to ∼1700 MU in comparison to P*_*narG*_*. The promoter activity for P*_*nasD*_* also reached a maximum prior to CDW_max_ and a slight trend was observed with an increase in activity at higher glucose levels. In addition, the decrease in activity for P*_*narG*_* was observed earlier during cultivation, while the maximum activity of P*_*nasD*_* was achieved later. This would also explain the nitrite peak observed and is in agreement to the results of Nakano et al. (1998a), who reported that the presence of nitrite along with the global regulator ResDE stimulates the expression of *nasD*. In this sense, nitrate first has to be reduced, and the nitrite produced stimulates *nasD* expression.

To sum up, the serum flask cultivations illustrated that the growth rate was reduced with increasing glucose concentration and that the promoter activity of P*_*narG*_* declined as well during the time course of cultivation. However, the experiments have also demonstrated that the cell density increased the more glucose was added. In addition, results revealed that a high amount of glucose is converted into acetate, while lactate can be considered as a neglectable metabolite in this experimental set-up. Furthermore, acetate production was not lower with less glucose present in the medium. The synthesis of acetate might also be needed to generate ATP, although nitrate respiration is the most efficient alternative respiratory mechanism compared to aerobic cultivations with oxygen as electron acceptor (Strohm et al., 2007).

### Batch vs. Fed-Batch Bioreactor Cultivation

**FIGURE 4:**
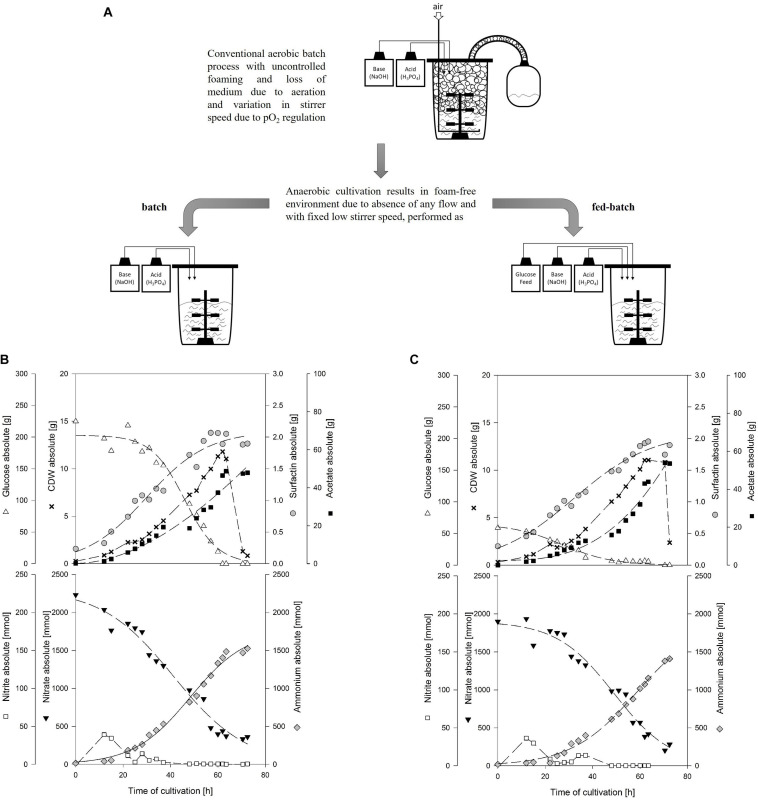
Diagrammatic representation of conventional aerobic batch process with foaming and anaerobic foam-free batch and fed-batch process **(A)**. Time course of 20 kg anaerobic bioreactor cultivation with strain *B. subtilis* JABs24 in a medium containing 0.1 mol/L NO_3_^–^ and 0.001 mol/L NH_4_^+^ performed as **(B)** batch with 10 g/L glucose and **(C)** fed-batch with 2.5 g/L glucose employing a feed with 600 g of a 25% glucose solution.

To further evaluate the impact of various glucose concentrations, the aim was to perform a batch bioreactor cultivation with strain *B. subtilis* JABs24 employing 10 g/L glucose and a fed-batch cultivation with an initial glucose concentration of 2.5 g/L glucose. A diagrammatic representation of the processes performed as well as the advantage over aerobic batch cultivations is given in Figure 4A. For the fed-batch process, an exponential feed phase which matches the glucose added in the batch cultivation was performed to evaluate the impact of a constantly low glucose concentration on growth, surfactin and acetate production.

Figure 4B (batch) and Figure 4C (fed-batch) illustrate the absolute values for cell dry weight, glucose, surfactin and acetate, as well as the absolute values of the anaerobic respiration metabolites nitrate, nitrite and ammonium for the two process strategies applied. All important process results as well as calculated yields are furthermore summarized in Table 4. For comparison, results of the reference serum flask cultivation employing 10 g/L glucose and yields of further non-conventional cultivation strategies to produce surfactin reported in literature are given as well.

**TABLE 4:** Summary of the results and calculated yields for the batch and fed-batch bioreactor process in comparison to a reference serum flask cultivation, as well as comparison to process parameters and yields obtained in different cultivation strategies reported.

	This study	This study	This study	Willenbacher et al. (2015a)	Chtioui et al. (2012)	Coutte et al. (2010)	Davis et al. (1999)
*B. subtilis* strain	JABs24	JABs24	JABs24	DSM 10^T^	ATCC 21332	ATCC 21332	ATCC 21332
Cultivation strategy	Batch bioreactor anaerobic	Fed-batch bioreactor anaerobic	Serum flask 10 g/L glucose anaerobic	Batch bioreactor 2.5/10 g/L glucose anaerobic	Rotating disk bioreactor aerobic	Membrane bioreactor aerobic	Batch bioreactor Oxygen-depleted nitrate-limited
Medium	20 kg	20 kg	0.1 L	1.0 L	1.2 L	3 L	1 L
CDW_max_ [g]	11.81	11.07	x	0.320*/0.586*	3.75	9.3	6.0*
CDW_max_ [g/L]	0.62	0.57	0.492	0.320/0.586	3.125*	3.1*	6.0*
CDW_max_ [h]	62	63.5	69	48*/140*	72	30*	26*
Surfactin at CDW_max_ [g]	1.89	1.95	x	0.06*/0.17*	0.254	0.727	0.350*
Surfactin at CDW_max_ [g/L]	0.100	0.101	0.190	0.06*/0.17*	0.212*	0.242*	0.350*
μ max [1/h]	0.112 (15 h)	0.093 (22 h)	0.109 (12 h)	0.105/0.074	x	x	x
*Y*_P/X_ max [g/g]	0.170 (15 h)	0.261 (12 h)	n.d.	x	x	x	x
*Y*_X/S_ max [g/g]	0.164 (37 h)	0.193 (31 h)	n.d.	x	x	x	x
*Y*_P/S_ max [g/g]	0.024 (54 h)	0.040 (25 h)	n.d.	x	x	x	x
*q*_surfactin_ max [g/(g⋅h)]	0.057 (15 h)	0.023 (22 h)	n.d.	x	x	x	x
overall μ [1/h]	0.062	0.056	0.041	0.049*/0.022*	0.064*	0.154*	0.203*
overall *Y*_P/X_ [g/g]	0.140	0.150	0.344	0.278/0.259	0.068	0.078*	0.075
overall *Y*_X/S_ [g/g]	0.051	0.064	0.044	0.120/0.049	0.189	0.164*	0.316*
overall *Y*_P/S_ [g/g]	0.007	0.010	0.015	0.033/0.011	0.013	0.013	0.018*
overall *q*_surfactin_ [g/(g⋅h)]	0.004	0.005	0.005	0.005/0.002	0.001*	0.002*	0.003*
Acetate [g/L] at CDW_max_	2.46	2.27	2.17	x	x	x	x
L-Lactate [g/L] at CDW_max_	0.16	0.19	0.24	x	x	x	x

*calculated or estimated within this study or previous published works of Willenbacher et al. (2015a) based on the data or figures given in the respective study.

The glucose concentration in the batch process was depleted after 62 h of cultivation and the CDW_max_ was reached at this time with 11.81 g (OD_600_ of 2.35). For the fed-batch process, the feed was started after 37 h of cultivation with a set growth rate of 0.04 1/h and an initial feed rate of 0.01 kg/h. This growth rate was chosen based on the results of the serum flask cultivations and was expected to not cause glucose accumulation during the feed phase. The glucose concentration at feed start was 0.61 g/L and maintained in a range of 0.31 ± 0.04 g/L during the feed phase. The CDW_max_ before glucose consumption was 11.07 g (OD_600_ of 2.15). For both cultivations and similar to the serum flask cultivations, cell density dropped after glucose depletion. According to Espinosa-de-los-Monteros et al. (2001) cell lysis occurred after the growth phase instead of sporulation. The surfactin concentration in both cultivations increased throughout cultivation and at CDW_max_, absolute values of 1.89 g and 1.95 g were reached. Consequently, both process strategies resulted in comparable surfactin titers and glucose limitation neither improved nor impaired surfactin productivity.

Analysis of nitrate revealed that there was no limitation and the reduction of nitrate as well as the increase in ammonium until the end of cultivation indicated that nitrate respiration occurred until growth stopped. At CDW_max_, 439.41 mmol and 417.30 mmol NO_3_^–^ were present in the medium for the batch and fed-batch process, respectively. Considering the results of both bioreactor and the serum flask cultivations, the nitrate demand for anaerobic nitrate respiration can be calculated as ∼150 mmol (NO_3_^–^)/g (CDW). Ammonium increased constantly up to 1403.49 and 1153.00 mmol until CDW_max_ was reached. For both cultivations, nitrite peaked at the beginning of cultivation and a second low nitrite peak was observed which actually occurred in a phase with reduced growth. After 48 h of cultivation nitrite was below 1 mmol. The highest concentration of nitrite was measured for both experiments after 12 h of cultivation with 39.13 mmol for the batch and 36.41 mmol for the fed-batch process. These observations match the data obtained from the serum flask cultivations where a nitrite peak was observed as well.

With respect to acetate, both processes showed a drastic increase in acetate with more than 40 g at CDW_max_. The acetate concentration increased almost parallel to the biomass and a significant difference in between the two process strategies was not observed. The overall *Y*_acetate/X_ and the *Y*_acetate/S_ for both batch and fed-batch process were in a similar range at CDW_max_ and reached 3.920 g/g and 0.206 g/g for the batch, and 3.961 g/g and 0.260 g/g for the fed-batch cultivation, respectively. The results obtained are also in agreement with the serum flask cultivations and illustrate that a lower glucose concentration even below 0.31 g/L throughout cultivation did not result in lower acetate production.

In comparison to acetate, lactate was produced as minor by-product in both cultivations with less than 4 g at CDW_max_. In a study performed by Espinosa-de-los-Monteros et al. (2001), authors reported that acetic acid and acetoin accumulated in cultivations with excess nitrate, whereas lactate and butanediol were produced when nitrate became limiting due to the presence of excess reduced cofactors. This would be in accordance to the current study, as nitrate decreased from 0.1 mol/L to ∼0.02 mol/L, but further investigations regarding these findings are necessary. Contrariwise, Cruz Ramos et al. (2000) reported a production of 23.3 mmol/L lactate and 16.4 mmol/L acetate cultivating strain *B. subtilis* 168 with nitrate as electron acceptor. However, they reported that the presence of nitrate reduced the formation of lactate and increased the production of acetate. In comparison to the current study, 50 mmol/L glucose and 50 mmol/L pyruvate were used as carbon source, which makes a comparison difficult, as the influence of pyruvate is not known. In addition, only 10 mmol/L NO_3_^–^ were used. This, based on the results of the current study, is not sufficient to ensure nitrate respiration throughout the cultivation in the presence of these amounts of carbon source. Consequently, it might be that the cultivation switched from nitrate respiration to fermentative growth and by that lactate was produced. This would also be in agreement with another statement made by Cruz Ramos et al. (2000) namely that the presence of nitrate actually represses the transcription of lactate dehydrogenase *ldh* and acetolactate synthase *alsS* genes.

Regarding the yields and process parameters, the fed-batch process reached higher maximum and overall yields *Y*_P/X_, *Y*_X/S_ and *Y*_P/S_. This is in agreement with the results of different glucose concentrations tested, as lower glucose levels led to an improved *Y*_X/S_ and *Y*_P/S_ and this is hence also valid for a feed phase. However, the employment of a fed-batch process did result neither in a better biomass or surfactin production, nor in a significantly lower acetate or lactate formation. Interestingly, in comparison to the serum flask cultivation, the surfactin concentration in both bioreactor cultivations reached only 0.100 g/L and 0.101 g/L, while 0.190 g/L surfactin was produced in the serum flask with less biomass. This is also illustrated by the *Y*_P/X_, which is more than double as high with 0.344 g/g in the serum flask. This observation is also in agreement with the *Y*_X/S_, indicating a better glucose conversion into biomass in the bioreactor cultivation in comparison to the serum flask, while the *Y*_P/S_ is superior in the serum flask cultivations.

To conclude, the fed-batch and batch cultivations showed that cell growth was observed as long as glucose was present in the medium, illustrating the general feasibility of anaerobic cultivations with strain *B. subtilis* JABs24 for the production of surfactin. Obviously, the next step in bioreactor process development would be to elongate the feed phase. In addition, neither process showed a significant superiority indicating that the initial glucose does not influence the overall performance of the cultivation. However, acetate production was also not reduced at lower glucose levels which makes this metabolite another interesting candidate for further investigations regarding its impact on anaerobic growth.

### Impact of Various Acetate Concentrations on Anaerobic Growth and Effect on Promoter Activity of P_*narG*_ and P_*nasD*_

During the previous cultivations, nitrite was shown to be reduced during the time course of cultivation even at an initial concentration up to 10 mmol/L, while acetate was detected in high amounts and the concentration curve was almost parallel to the growth curve with final acetate concentrations up to 2.5 g/L. Under aerobic conditions, the production of acetate is often correlated to overflow metabolism (Presecan-Siedel et al., 1999; Kabisch et al., 2013). In addition, *B. subtilis* is not able to grow on acetate aerobically due to the absence of genes of the glyoxylate shunt (Kabisch et al., 2013). However, under anaerobic fermentative conditions, acetate synthesis is important for generation of energy because acetate synthesis goes along with the AckA dependent formation of ATP (Cruz Ramos et al., 2000). Under anaerobic respiratory conditions, no distinct hypothesis was found that explains the acetate formation under nitrate respiratory conditions. The utilization of nitrate as alternative electron acceptor is reported to be the most favorable in view of ATP yield (Marino et al., 2001). Nevertheless, acetate is also often declared as growth inhibiting substance, but the effect varies in between different bacterial species as demonstrated by Lasko et al. (2000) testing *E. coli*, *Acetobacter aceti*, *Staphylococcus capitis*, *Gluconobacter suboxydans*, *Lactobacillus acetotolerans*, and *L. bulgaricus* in aerobic cultures. In another study, addition of 128 mmol/L acetate resulted in a reduced growth rate from 0.75 1/h to 0.4 1/h in an *E. coli* culture at pH 7.4 (Pinhal et al., 2019). However, little information is available on the effect of acetate on *B. subtilis*. Studies dealing with acetate and declaring growth inhibiting effect mostly refer to experiments with *E. coli*.

**FIGURE 5:**
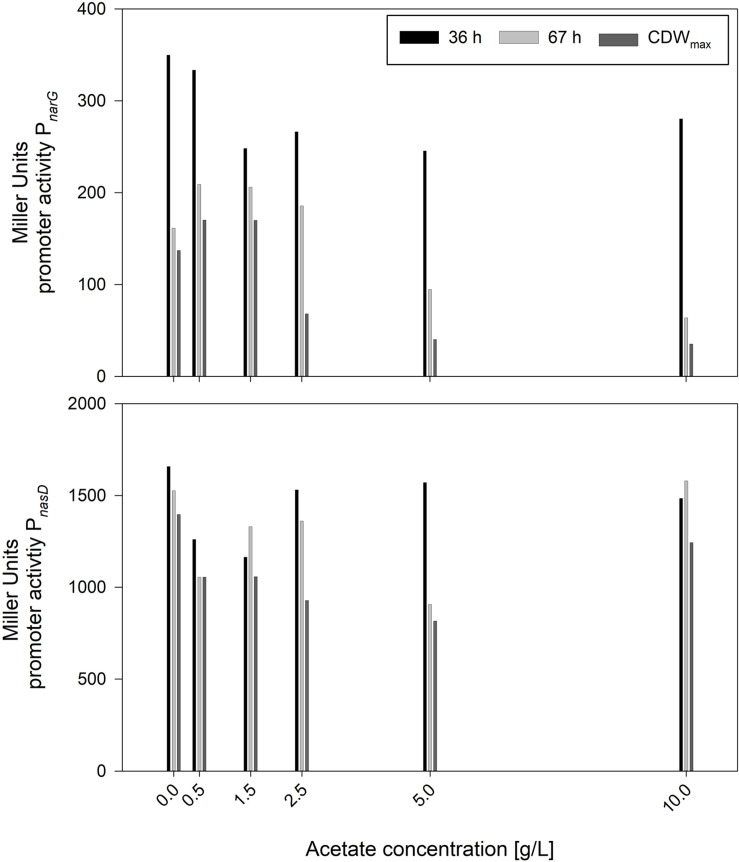
Influence of different acetate concentrations on the expression of P*_*narG*_* and P*_*nasD*_* after 36 and 67 h of cultivation and at CDW_max_ employing strains *B. subtilis* MG1 and MG5.

In contrast to nitrite, the concentration of acetate was increasing steadily. Hence, serum flask cultivations were performed with various initial acetate concentrations. Other than for nitrite, cultivations were performed directly with 10 g/L glucose to monitor effects in longer cultivations. The cultivation results with strains JABs24, MG1 and MG5 as triplicate are given in Table 5. The maximum CDWs were in a similar range and varied in between 0.465 and 0.532 g/L and no correlation was found between initial acetate concentration and CDW_max_. The time to reach CDW_max_ was ∼69 h for the reference and ∼105 h for 10 g/L acetate added. In this sense, acetate has an influence comparable to that of nitrite with respect to the time of cultivation to reach CDW_max_. However, while μ_max_ for nitrite was in an overall similar range (Figure 2), μ_max_ for acetate was lower the more acetate was added. This indicates that acetate did not prolong the lag-phase such as demonstrated for nitrite but has an overall negative effect on cellular growth. Furthermore, the overall growth rate μ was reduced from 0.041 ± 0.000 1/h to 0.029 ± 0.001 1/h applying 0 g/L and 10 g/L acetate, respectively. Due to the similar final CDW values obtained, the *Y*_X/S_ for all cultivations was as expected in the same range and the mean yield was determined as 0.042 ± 0.004 g/g. Hence, added acetate did not reduce biomass formation. With respect to the acetate produced on top of the initial concentration, the reference cultivation reached the lowest values, while for the other cultivations a slight overall increase was detected. In accordance to this slight increase, also the yield *Y*_acetate/X_ showed this effect with the same significant change in between 0 g/L and 0.5 g/L acetate added. To further illustrate the trend of the activities of both P*_*narG*_* and P*_*nasD*_*, the data after 36 h and 67 h of cultivation, as well as at CDW_max_, are illustrated in Figure 5. In general, the activity decreased during the time course of cultivation within the different acetate concentrations tested. In regard to the promoter activity at CDW_max_, a reduction in activity was observed for P*_*narG*_* and Miller Units decreased from 137 MU for 0 g/L acetate to 35 MU in the cultivation with 10 g/L acetate added. Considering the activity of P*_*nasD*_*, values were as previously reported much higher, and except for 10 g/L the activity at CDW_max_ was also reduced from 1397 MU for the reference and 815 MU employing 5 g/L. Interestingly, considering the percentage change, the decrease in activity was overall more severe for the nitrate reductase and further studies regarding the negative effect of either acetate directly or for example the onset of nitrate limitation as mentioned before on the reduced activity must be performed.

**TABLE 5:** Summary of effect of different acetate concentrations on anaerobic growth.

*c*_acetate_ [g/L]	0	0.5	1.5	2.5	5	10
CDW_max_ [g/L]	0.492 ± 0.010	0.470 ± 0.049	0.470 ± 0.033	0.474 ± 0.025	0.532 ± 0.011	0.465 ± 0.011
*μ*_max_ [1/h]	0.123 ± 0.020	0.095 ± 0.005	0.083 ± 0.013	0.072 ± 0.009	0.076 ± 0.002	0.069 ± 0.006
Overall μ [1/h]	0.041 ± 0.000	0.042 ± 0.004	0.037 ± 0.002	0.033 ± 0.003	0.039 ± 0.001	0.029 ± 0.001
Δ*c*_acetate_ [g/L]	2.152 ± 0.249	2.689 ± 0.222	2.850 ± 0.108	3.014 ± 0.226	3.143 ± 0.372	2.987 ± 0.438
*Y*_acetate/X_ [g/g]	4.386 ± 0.557	5.742 ± 0.251	6.084 ± 0.234	6.386 ± 0.686	5.906 ± 0.621	6.405 ± 0.813
*Y*_acetate/S_ [g/g]	0.202 ± 0.020	0.262 ± 0.018	0.244 ± 0.020	0.271 ± 0.014	0.276 ± 0.047	0.265 ± 0.037

To sum up, the inhibitory effect of acetate was more pronounced than for ammonium and especially nitrite, as acetate synthesis occurred throughout growth and concentrations did not decrease as shown for nitrite. High acetate concentrations reduced both the overall growth rate as well as the maximum growth rate and also the expression of *narG* and *nasD* were shown to be affected. Indeed, it is of upmost interest to further study this effect. These observations are counterintuitive, as acetate formation results in ATP supply, but in the same time its accumulation led to notable growth inhibition. Further studies regarding the role of acetate, e.g., by deleting the genes involved in acetate synthesis, namely acetate kinase *ackA* and phosphate acetyltransferase *pta*, displays one option. Regarding this approach, Cruz Ramos et al. (2000) already reported that a *B. subtilis* Δ*pta* mutant strain produced less acetate in the presence of nitrate, but growth was also drastically reduced, indicating the importance of this metabolic pathway for anaerobic growth by nitrate respiration. Still, strains unable to produce acetate or able to utilize acetate anaerobically might be an interesting alternative for surfactin production processes. For example, a *B. subtilis* strain carrying the glyoxylate shunt genes from *B. licheniformis* DSM 13 was reported to be more robust and showed a better growth aerobically (Kabisch et al., 2013). Longing for more robust strains able to grow anaerobically and synthesize surfactin without acetate accumulation clearly presents an opportunity for further strain engineering. Even if growth rates are lower, but productivity is maintained throughout a prolonged time window, a significantly increased product titer could be achieved in a foam-free environment.

## Summarizing Remarks and Further Considerations for Future Research

The results presented illustrate the feasibility of an anaerobic nitrate respiration process which also lays the basis for the establishment of other processes where either foaming is a major issue, or the target product is sensitive towards oxygen.

The ability of *B. subtilis* to grow anaerobically was reported in previous studies. Table 6 summarizes different studies with the respective medium, carbon and nitrogen sources used and OD-values achieved.

**TABLE 6:** Comparison of anaerobic cultivations with *B. subtilis* in literature with the medium used and OD-values reached.

Strain	Medium	Condition	Carbon source [g/L]	Nitrogen source [mmol/L]	OD_max_	References
*B. subtilis* JABs24	Defined (modified Cooper’s	anaerobic	Glucose: 10	NO_3_^–^: 100	1.85 (serum flask)	This study
(derived from 168)	mineral salt medium)			NH_4_^+^: 1	2.35 (bioreactor)	
*B. subtilis* DSM 10^T^	Defined (modified Cooper’s	anaerobic	Glucose: 10	NO_3_^–^: 117.7	1.76	Willenbacher et al. (2015a)
	mineral salt medium)			NH_4_^+^: 100		
*B. subtilis* DSM 10^T^	Defined (modified Cooper’s	anaerobic	Glucose: 7.5	NO_3_^–^: 117.7	2.568	Willenbacher et al. (2015a)
	mineral salt medium)			NH_4_^+^: 100		
*B. subtilis* LCB6	Defined (Spizizen’s	aerobic	Glycerol: 10 mL/L	NO_3_^–^: 24	∼2	Clements et al. (2002a)
(derived from I168)	minimal medium)	anaerobic		NH_4_^+^: 30	∼0.1	
*B. subtilis* LCB6	Defined (Spizizen’s	aerobic	Glucose: 10	NO_3_^–^: 24	3	Clements et al. (2002b)
(derived from I168)	minimal medium)	anaerobic		NH_4_^+^: 30	0.12	
*B. subtilis* 168	Defined (minimal medium)	anaerobic	Glucose: 9	NO_3_^–^: 10	1	Cruz Ramos et al. (2000)
			Pyruvate: 4.4	NO_2_^–^: 10	0.7	
				x	1	
*B. subtilis* JH642	Complex (LB with	anaerobic	Glucose: 0.18	NO_3_^–^: 10	1.1	Hoffmann et al. (1998)
	supplements)			NH_4_^+^: 4		
				NO_2_^–^: 10	1	
				NH_4_^+^: 4		
				x	0.7	
*B. subtilis* JH642	Defined (minimal medium)	aerobic	Glucose: 9	x	10	Marino et al. (2001)
		anaerobic		NO_3_^–^: 10	1.1	
				NO_2_^–^: 10	1.05	
				x	1	

Next to fundamental research on anaerobic growth, several studies used this approach to synthesize lipopeptides, and in this case surfactin (Javaheri et al., 1985; Davis et al., 1999; Willenbacher et al., 2015a; Geissler et al., 2019b). This process strategy results in promising *Y*_P/X_ values, meaning less biomass waste is produced per gram surfactin, and, which is an important aspect for operating and process control, foaming is completely avoided. This also allows using the full capacity of a bioreactor which leads to an improvement in the volumetric productivity, while the volume in foaming processes is often reduced so that the foam can accumulate in the headspace (St-Pierre Lemieux et al., 2019). Foam-free strategies also make the addition of antifoam agents and the implementation of foam breakers, which results in high energetical input, needless. Furthermore, both techniques to degrade foam can result in cellular stress and consequently reduced productivity (St-Pierre Lemieux et al., 2019). Another advantage of anaerobic cultivations is that the stirrer speed can be kept throughout the process, while pO_2_ regulation generally goes along with increasing aeration rates and stirrer speeds. Both parameters significantly influence foaming and as the process values increase with increasing biomass, also foam formation is enhanced (St-Pierre Lemieux et al., 2019).

However, several concluding remarks should be mentioned that need to be addressed in further studies, especially as the target product surfactin reached much lower concentrations in the bioreactor cultivations. One issue faced was the pH value. Ideally, also as demonstrated by Willenbacher et al. (2015a), the anaerobic cultivation leads to a basic pH shift. During that cultivation, nitrogen airflow was adjusted above the liquid level, with the aim to reduce the backflow of oxygen from the air. In the current study, the pH shifted to acidic conditions and hence base needed to be added to maintain the pH. In preliminary bioreactor cultivations, different nitrogen flows were tested. Indeed, employing a nitrogen flow through the medium resulted in a decrease in pH, but also resulted in foaming. Employing a nitrogen flow above the liquid level was also not sufficient, which illustrates the difficulties in transferring results from a 1 L bioreactor cultivation as in Willenbacher et al. (2015a) to a 20 kg cultivation as in this study. The decrease in pH was assumed to be both due to the production of acidic products such as acetate, and due to the accumulation of CO_2_ in the medium which converts to H_2_CO_3_ (Killam et al., 2003). Although acetate was not measured by Willenbacher et al. (2015a), it is presumably to assume that acetate was produced due to similarities in e.g. CDW, and hence the effect of the acidic pH shift in the current study is most likely due to accumulated CO_2_. Nevertheless, as the data for surfactin and CDW reported by Willenbacher et al. (2015a) and Geissler et al. (2019b) are well in accordance to the results obtained in this study, it can be hypothesized that there is no pivotal negative effect when CO_2_ is not stripped.

The anaerobic growth of *B. subtilis* is considered an interesting research field and many studies deal with fundamental research on sequencing, cloning or regulatory mechanisms (Cruz Ramos et al., 2000; Clements et al., 2002a, b). Consequently, many efforts are needed to improve the production process to further improve the yields. As summarized by Geissler et al. (2019a), there are three methods to improve the titers, namely medium and process parameter optimization, strain engineering and establishing process strategies. For example, addition of further carbon sources, amino acids or vitamins was reported to improve anaerobic growth (Carvalho et al., 2010; Javed and Baghaei-Yazdi, 2016). In terms of process strategies, appropriate feed profiles must be established to, for example, maintain a certain glucose/nitrate-ratio which influences the synthesis of metabolic by-products such as acetate and lactate (Espinosa-de-los-Monteros et al., 2001). The incorporation of a short aerobic phase displays an interesting option as this resulted in faster growth as reported by Cruz Ramos et al. (2000) and might be beneficial when aerobic pre-cultures are used as performed in this and many other studies. However, on the contrary, Willenbacher et al. (2015a) employed anaerobic pre-cultures and in comparison to the results of Geissler et al. (2019b) no significant differences were observed that could be attributed to the pre-cultures. The third strategy, strain engineering, poses another option to improve the anaerobic growth and the surfactin synthesis of *B. subtilis*. In this field, promoter exchange or gene knockout studies display a promising approach. As illustrated in the previous results, the synthesis and accumulation of acetate was pointed out as bottleneck and is hence a starting point for future studies.

## Conclusion

The present study demonstrated the applicability of anaerobic foam-free processes by nitrate respiration for the synthesis of surfactin in a *B. subtilis* cultivation. Nevertheless, even at low substrate concentrations, significant production of acetate could be observed. As such, acetate was identified as a target metabolite for ongoing research and strain development. Furthermore, future studies should investigate the reported decrease in the promoter activity P*_*narG*_* during the time course of cultivation as well as its decrease in the presence of high ammonium and acetate concentrations. Concluding, this study constitutes an important step towards the development of longer, more robust and more productive processes with *B. subtilis* using anaerobic nitrate respiration.

## Data Availability Statement

The original contributions presented in the study are included in the manuscript/Supplementary Material, further inquiries can be directed to the corresponding author.

## Author Contributions

MHo planned and executed the experiments, collected data, created the graphs, and drafted the whole manuscript. DF, LS, and KR performed part of the experiments and collected and evaluated corresponding data. SX under supervision of KH constructed the reporter strains. LL supported in interpretation of results. MHe contributed to conception and design of the study and interpretation of the experiments. RH substantially contributed to conception and design of the conducted experiments. All authors read and approved the final version of the manuscript.

## Conflict of Interest

The authors declare that the research was conducted in the absence of any commercial or financial relationships that could be construed as a potential conflict of interest.
