# Supplementary material for: Towards the Anaerobic Production of Surfactin Using Bacillus subtilis
Source: Front Bioeng Biotechnol. 2020 Nov 26;8:554903. doi: 10.3389/fbioe.2020.554903 (PMC7726195; doi:10.3389/fbioe.2020.554903)

**Supplementary Material**

Towards the Anaerobic Production of Surfactin Using *Bacillus subtilis*

**Mareen Hoffmann, Diana Stephanie Fernandez Cano Luna, Shengbin Xiao, Lars Stegemüller, Katharina Rief, Kambiz Morabbi Heravi, Lars Lilge, Marius Henkel^*^, Rudolf Hausmann**

*Department of Bioprocess Engineering (150k), Institute of Food Science and Biotechnology (150), University of Hohenheim, Stuttgart, Germany*

*** Correspondence:**

Marius Henkel

Marius.henkel@uni-hohenheim.de

**Table S1** Oligonucleotides used in this study.

| Name | Sequence (5’ → 3’) | Application |
| --- | --- | --- |
| s1001 | AAAAAAACCGGTACCATGAACTTTTTGGCTATGCTTCAGC | Amplification of *narG* promoter region (*Age* I or *Nde* I restriction site) |
| s1002 | AAAAAACATATGAGTTCACTCCTTCCGAGTCAGG |  |
| s1011 | AAAAAAACCGGTACCCGCGGGGAAGCATCACCGTC | Amplification of *nasD* promoter region (*Age* I or *Nde* I restriction site) |
| s1012 | AAAAAACATATGATGATCCGCTCCTTATCAAATGT |  |
| s7406 | AAAAAAGGATCCGAATAATCTGCGTAATAGACTTTC | Confirmation of *amyE* disruption |
| s7409 | AAAAAAGGATCCACACCGCCGACATTCGC |  |

**S2** Overview of equations used to calculate the process parameter.

| Overall biomass per substrate yield | $\mathrm{overall}Y_{X/S}= \frac{m_{CDW max}- m_{CDW 0h}}{m_{glucose 0h}- m_{glucose CDW max}}$ |
| --- | --- |
| Biomass per substrate yield | $Y_{X/S}= \frac{\Delta m_{\mathrm{CDW}}}{\Delta m_{\mathrm{glucose}}}$ |
| Overall product per biomass yield | $\mathrm{overall} Y_{P/X}= \frac{m_{surfactin CDW max}-m_{surfactin 0h}}{m_{CDW max}}$ |
| Product per biomass yield | $Y_{P/X} = \frac{{\Delta m}_{\mathrm{surfactin}}}{\bar{m}_{\mathrm{CDW}}}$ |
| Overall specific productivity | ${\mathrm{overall}q}_{spec.,surfactin}= \frac{{\mathrm{overall} Y}_{P/X}}{\Delta t}$ |
| Specific productivity | $q_{spec.,surfactin} = \frac{Y_{P/X}}{\Delta t}$ |
| Overall product per substrate yield | $\mathrm{overall}Y_{P/S}= \frac{m_{surfactin CDW max}-m_{surfactin 0h}}{m_{glucose 0h}- m_{glucose CDW max}}$ |
| Product per substrate yield | $Y_{P/S} = \frac{\Delta m_{\mathrm{surfactin}}}{\Delta m_{\mathrm{glucose}}}$ |
| Overall growth rate | $\mathrm{overall} \mu= \frac{\ln\frac{m_{CDW max}}{m_{CDW 0h}}}{t_{CDW max}- t_{CDW 0h}}$ |
| Specific growth rate | $\text{µ} = \frac{\ln\frac{m_{\mathrm{CDW}_{2}}}{m_{\mathrm{CDW}_{1}}}}{t_{2}- t_{1}}$ |

**Figure S3** Miller Units measured during cultivations of *B. subtilis* reporter strains MG1 (P*_narG_*-*lacZ*) and MG5 (P*_nasD_*-*lacZ*) with 7.5 g/L or 10 g/L glucose and varying ammonium concentrations.


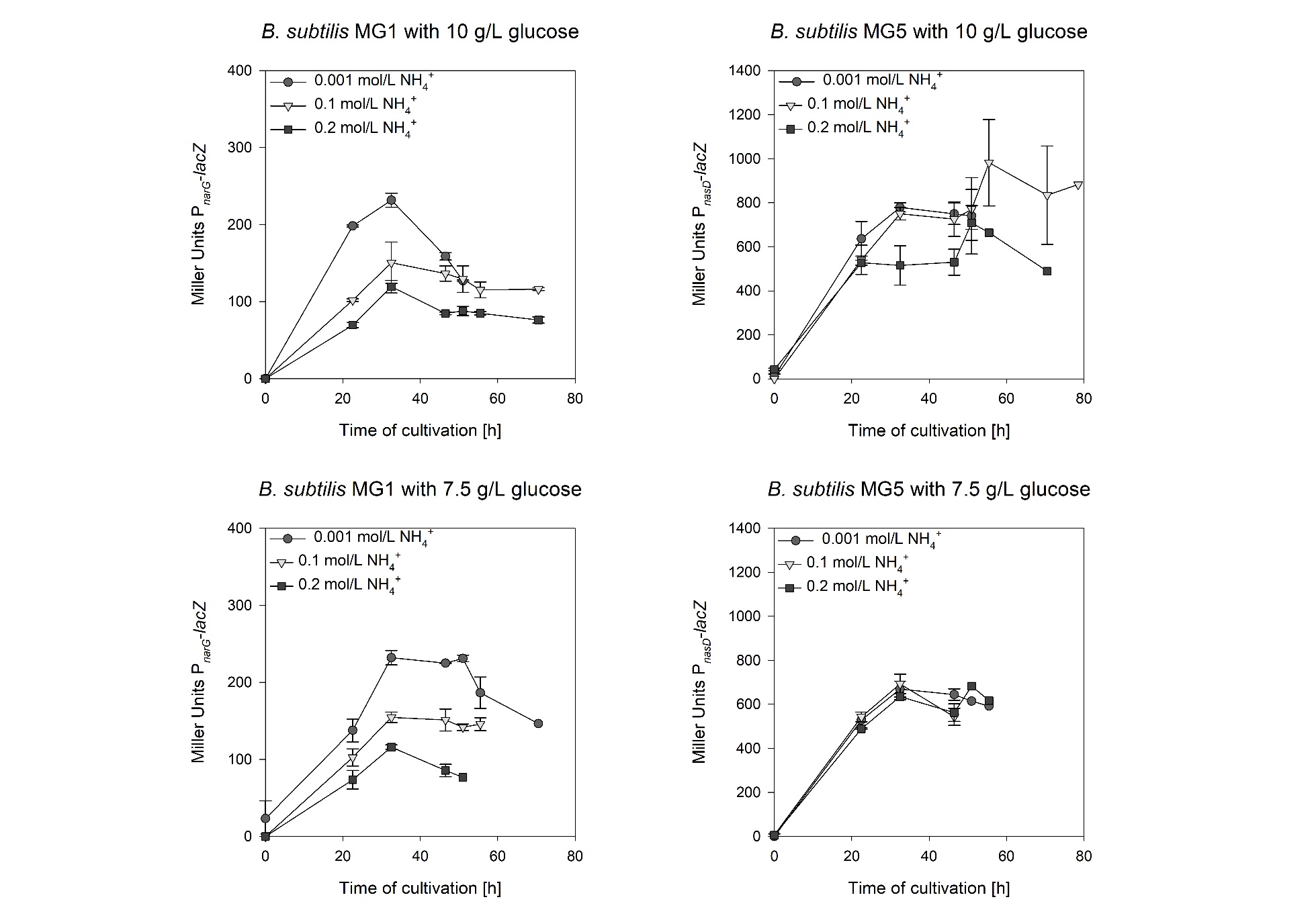

Supplement: Supplementary file 1 [file Data_Sheet_1.docx]
